# Awareness of school students on sexually transmitted infections (STIs) and their sexual behavior: a cross-sectional study conducted in Pulau Pinang, Malaysia

**DOI:** 10.1186/1471-2458-10-47

**Published:** 2010-01-30

**Authors:** Mudassir Anwar, Syed Azhar S Sulaiman, Keivan Ahmadi, Tahir M Khan

**Affiliations:** 1 School of Pharmacy and Health Sciences, International Medical University, Kuala Lumpur, Malaysia; 2 School of Pharmaceutical Sciences, Universiti Sains Malaysia, Pulau Pinang, Malaysia; 3 School of Pharmacy, Island College of Technology, Sungai Rusa, 11000 Balik Pulau, Pulau Pinang, Malaysia

## Abstract

**Background:**

Sexually transmitted Infections (STIs) rank among the most important health issues for the people especially the young adults worldwide. Young people tend to engage in sexual activity at younger ages in the past decade than in the 1970s, and 1980s. Knowledge is an essential precursor of sexual risk reduction. A cross-sectional study was conducted in Pulau Pinang, Malaysia, to produce the baseline information about school students' awareness and perception about sexually transmitted Infections (STIs) and their sexual activity to help establish control and education programmes.

**Methods:**

Students from form 4 (aged between 15 to 16 years), form 5 (aged between 16 to 17 years) and form 6 (aged between 18 to 20 years) in their class rooms were approached and asked to complete self administered and anonymous pre-validated questionnaires. SPSS for windows version 13 was used to analyze the results statistically and results were presented in tabular form.

**Results:**

Data was collected from 1139 students aged between 15 to 20 years, 10.6% of which claimed that they never heard about STIs. Sexual experience related significantly with gender, race, and education level. Approximately 12.6% claimed to have sexual experience of which 75.7% had their sexual debut at 15-19 years and 38.2% were having more than 3 partners. Sexual experience was found to be significantly associated with gender (*p *= 0.003), ethnicity (*p *= 0.001) and education level (*p *= 0.030). However, multiple partner behaviour was significantly associated only with gender (*p *= 0.010). Mean knowledge score was 11.60 ± 8.781 and knowledge level was significantly associated with religion (p = 0.005) education level (*p *= 0.000), course stream (*p *= 0.000), socioeconomic class (*p *= 0.000) and sexual experience (*p *= 0.022).

**Conclusions:**

It was concluded that school students have moderate level of knowledge about STIs although they are sexually active. Interventions such as reinforcing the link between STIs and HIV/AIDS, assessing the current status of sexuality education in schools and arranging public talks and seminars focusing on STIs prevention education are needed to improve their awareness.

## Background

Sexually transmitted Infections (STIs) rank among the five most important causes of healthy productive life loss in developing countries [1]. The World Health Organization (WHO) estimates that the global incidence of new cases of selected curable STIs - Gonorrhea, Syphilis, Chlamydia and Trichomoniasis was 340 million in 1999. The largest number of new infections occurred in South and Southeast Asia [2].

According to a report published by UNAIDS/WHO working group on global HIV/AIDS and STI surveillance in December 2006 there were 70,559 reported cases of STIs in Malaysia by the end of 2005 out of which 10,663 cases were of AIDS and the rest were of other STIs [3]. Evidence strongly suggested that presence of one STI facilitated transmission of HIV by a factor of 2-5 times [4,5].

Historically knowledge about STIs has been very low even in communities where there is high prevalence of STIs. Sometimes STIs may be viewed as unavoidable or may even be viewed as an "initiation into adulthood". There may be lack of concern about STIs because they may be viewed as easily curable [6]. Knowledge is an important prevention factor for STIs [7]. It has been suggested that knowledge about STI transmission might influence sexual behavior [8]. There is some data available on youth's knowledge on STIs in some of the states of Malaysia with Pulau Pinang as one of the exception. This research will serve as baseline information that could be linked with future follow-up studies or interventions. The main objectives of the study were to describe the level of knowledge and sexual behavior of school students and their attitudes towards STIs and to determine the association between socio-demographic factors and sexual practice with the knowledge towards STIs.

## Methods

This cross-sectional, descriptive study was conducted in Pulau Pinang during January 2005 to April 2005. Pulau Pinang is one of the fourteen states located in the northwest of Malaysia and comprises of the mainland and Penang Island. Its population is 1265.1 thousand [9]. Different ethnic groups inhabiting the state are Malays (42.5%), Chinese (46.5%), Indians (10.6%), and other minorities (0.4%) [9].

The stages of education system in Malaysia consist of pre-school, priAprily education, secondary education, tertiary education, and postgraduate studies. The education system is attained either through government schools or private schools mandated by Malaysian law and handled by the Ministry of Education. Secondary education comprises of 5 years of schooling known as form 1 to form 5 catering the education needs of children aged between 13 to 18 years. At the end of form 5 students are required to take the Sijil Pelajaran Malaysia (SPM) or the Malaysian Certificate of Education examination, before graduating secondary school. After form 5, students (aged between 18 to 20 years) can take up either matriculation (British 'A' levels, the Canadian matriculation etc) or form 6, comprising of two years of education before students could sit for Sijil Tinggi Persekolahan Malaysia (STPM) or Malaysian Higher School Certificate examination.

Convenience sampling technique was employed for the selection of schools in Penang Island. A total of eleven schools were approached to participate in this survey and the heads from six of the schools gave permission to conduct this study in their schools. All the students belonging to form 4, form 5 and form 6 (representing adolescents and young adults) of the six government schools were included in the study. Private schools in Malaysia mostly represent one out of the three main ethnic groups (Malays, Chinese and Indians). Majority of private schools are either representatives of Chinese population or Indian population. Therefore, in order to avoid bias only government schools were approached with the understanding that they represent all the three main ethnic groups according to their real distribution in the country.

For data collection an 18-item questionnaire was developed in English. The questionnaire was then translated into Bahasa Melayu (national language of Malaysia). Seven questions addressed socio-demographic information, one on the knowledge about different types of STIs, one on symptoms, one on preventive measures, one on the risk groups, one on complications, one on transmission route, one on causative organisms, six on sexual attitudes and history and one on knowledge source. Each knowledge question included multiple options or statements, for example the options for the question on different STIs that the respondents could be aware of included AIDS, syphilis, gonorrhoea, rheumatoid arthritis, herpes, leukemia, chlamydia, trichomoniasis and don't know. The respondents could select more than one option.

Ethical approval was obtained from the Social and Behavioural Research Ethics Committee, University Sains Malaysia. The face and content validity was done by the professionals at the Disciplines of Social and Administrative Pharmacy at University Sains Malaysia and by the Department of Biostatistics General Hospital Pulau Pinang. The reliability scale was applied to all the variables comprising the knowledge domain i.e Knowledge about the different STIs, symptoms, awareness about causes/prevention and diagnosis and therapy. The reliability and internal consistency of the tool were estimated on the basis of Cronbach's Alpha (= 0.81).

To collect data, the head masters of the schools were approached and were requested to give the permission for conducting the survey. Meetings with the students of form 4, form 5, and form 6 were arranged in their class rooms; verbal consent was taken from the students and instructions were given to fill the questionnaires. Students were asked to fold the questionnaire after filling, to maintain the privacy. All questionnaires were anonymous.

Each correct response under a question equaled one score. Maximum score that could be obtained was 33. Participants were evaluated according to the points they received from knowledge questions. Students who scored 0-10 points were designated as having low level of knowledge, 11-21 as moderate level of knowledge and 22-33 high level of knowledge. Results were analyzed statistically using Statistical Package for the Social Sciences (SPSS) for windows version (13.0). Comparisons among groups were made using appropriate inferential tests such as Student t-test, Chi square test, and ANOVA. Statistical significance level used was 0.05.

## Results

A total of 1194 students from form 4, form 5 and form 6 were surveyed. The responses of 1139 (95.4%) participants were valid; the rest of 55 (4.6%) survey forms were rejected because they were not completely filled by the participants. The socio-demographic characteristics of the students surveyed are shown in Table 1.

**Table 1:** Socio-demographic characteristics of the students surveyed

Characteristic	N	%
**Participants:**	1139	95.4
Male	474	41.6
Female	665	58.4
		
**Mean age (range):**	17.2 (15-20)	-
		
**Race:**		
Malay	576	50.6
Chinese	348	30.6
Indian	211	18.5
Others	4	0.4
		
**Religion:**		
Muslim	584	51.3
Hindu	180	15.8
Christian	78	6.8
Buddhist	293	25.7
Others	4	0.4
		
**Education level:**		
Form 4	339	29.8
Form 5	417	36.6
Form 6	383	33.6
		
**Course stream:**		
Arts	575	50.5
Science	564	49.5
		
**Socioeconomic status**		
Low (less than RM 1000)	445	39.1
Middle (RM 1000-RM 3000)	532	46.7
High (RM 3100 and above)	162	14.2

Mean knowledge score was found to be 11.60 ± 8.781. As expected students of form 6 were found to be more knowledgeable (mean score = 13.05 ± 8.369) than their counterparts. Similar expected results were obtained for the course stream whereby science stream students were found to be more knowledgeable (mean score = 12.60 ± 8.397) than arts stream students (mean score = 10.65 ± 8.758). Interestingly students who claimed to be sexually active were less knowledgeable (mean score = 11.49 ± 8.710) than those who never had sexual intercourse (mean score = 12.90 ± 8.479). Mean knowledge score was significantly associated with religion (*p *= 0.005) education level (*p *= 0.000), course stream (*p *= 0.000), socioeconomic class (*p *= 0.000) and sexual experience (*p *= 0.022). Table 2 shows the detailed findings on mean knowledge score.

**Table 2:** Comparison of scores on knowledge questions according to socio-demographic factors and sexual experience of school students.

N = 1139	n	Mean score ± SD	t-value/F-value	p-value
**Gender**				
Male	474	11.77 ± 8.991		
Female	665	11.50 ± 8.481	0.743*	0.458^†^
				
**Ethnicity**				
Malay	576	11.64 ± 8.505		
Chinese	348	11.42 ± 9.046	0.276**	0.843^†^
Indian	211	11.87 ± 8.660		
				
**Religion**				
Muslim	584	11.67 ± 8.516		
Hindu	180	11.43 ± 8.395	3.696**	0.005^‡^
Christian	78	13.87 ± 8.535		
Buddhist	293	11.07 ± 9.143		
				
**Education level**				
Form 4	339	10.24 ± 8.510		
Form 5	417	11.41 ± 8.736	20.922**	0.000^‡^
Form 6	383	13.05 ± 8.369		
				
**Course stream**				
Arts	575	10.65 ± 8.758		
Science	564	12.60 ± 8.397	-0.56*	0.000^‡^
				
**Socioeconomic status**				
Low (less than RM 1000)	445	10.92 ± 8.789		
Middle (RM 1000-RM 3000)	532	11.76 ± 8.292	7.739**	0.000^‡^
High (RM 3100 and above)	162	13.03 ± 9.350		
				
**Sexual experience**				
Yes	143	12.90 ± 8.479		
No	963	11.49 ± 8.710	-2.293*	0.022^‡^

* t-value (Student's t-test)

** F-value (ANOVA)

^‡ ^Significant (p < 0.05)

^† ^Non-significant (p > 0.05)

About 121 (10.6%) students surveyed claimed that they never heard about STIs. However, AIDS was found to be the most commonly known STIs among the students who claimed to have heard of STIs. For detailed findings regarding the students' knowledge on different aspects of STIs "see Additional file 1".

Approximately 12.6% claimed to have sexual experience while 2.8% did not respond to this question. Majority of students (75.7%) who claimed to have sexual experience had their sexual debut at ages 15-19 years. No significant difference was detected between male and female or among different ethnic groups, religions, education levels, course streams and socioeconomic classes when compared for age at sexual debut. It was also found that sexual experience (intercourse) was significantly associated with gender (*p *= 0.003), ethnicity (*p *= 0.001) and education level (*p *= 0.030). However multiple partner behavior was significantly associated only with gender (*p *= 0.010) whereby more male participants claimed to have multiple partners as compared to female participants "see Additional file 2".

## Discussion

### Sexual experience

Sexual activity was significantly associated to gender, with males being more sexually active as compared to the females. This finding correlates with the findings of Siti Norazah Zulkifli 2000 on sexual practices of unmarried youth, Kamarudin Bin Kana 2003 in a study on school students from Kelantan and Lee *et al*. 2006, in a study on school students from Negeri Sembilan [10-12]. A possible reason of the finding whereby students from from 4 were found to be more sexually active as compared to the students from form 6 may be that, as the students get more mature they tend to hide the reality and feel uncomfortable to share their personal experiences.

### Initiation of sexual activity

Sexual debut at 15-19 years, without any gender difference, suggests sexual drives at young ages may overcome moral issues [10,13]. This finding correlates with the finding of Lee *et al*. 2006, whereby the mean age at first sexual intercourse was 15 years [12].

### Multiple partner behavior

Multiple partner behavior was very common among the students surveyed and was significantly associated with gender. Having multiple sex partners is a significant behavioral risk factor for HIV/STIs [14]. These findings are consistent with studies in Papua New Guinea and Turkey [15,16]. Male participants were having more sexual partners as compared to the female participants. Again the reason for this finding may be the conservative nature of society where female feel uncomfortable to share their sexual experiences.

### STI knowledge

A high percentage of participants knew AIDS as an STI. However, the rate fell rapidly to unsatisfactory levels for other STIs. This signifies that the IEC (information, education and communication) programs focused or concentrated on AIDS with little or no emphasis given to other STIs. Although accurate knowledge alone is insufficient to produce changes in attitude and behavior, it is a necessary component towards a person's developing the motivation to change his or her behavior [17,18]. There were also some misconceptions as considerable number of students perceived leukemia and rheumatoid arthritis as STIs. Hingson *et al*. 1990 suggested that people's misconception and lack of knowledge about the major non-HIV STIs put them more at risk for consequences of their sexual behavior [19].

The findings of this study show that school students are also not sufficiently informed about symptoms, measures to avoid getting STIs, risk groups, complications and transmission routes of STIs. According to Johnson *et al*. 1999 correct knowledge about methods of preventing STIs is an essential starting point in the behavior change process for individuals who have misconceptions about behaviors that prevent STI infection. Such misconceptions may lead to the use of ineffective STI protective strategies in place of effective, but less acceptable strategies, for example douching instead of condom use [20].

### Knowledge difference

As expected and consistent with the findings of other studies, visual and print media and friends were the main sources of knowledge about STIs [21,22]. The fact that HIV infection and AIDS appear in the media most frequently in Malaysia may explain their high rating. These diseases also are frequently discussed in schools. However, other STIs do not get such attention and consequently, are not recognized. It was disappointing but not unexpected that families, teachers and text books were the uncommon sources of knowledge.

The overall mean score of 11.60 on knowledge questions shows that school students have moderate level of knowledge. As expected, education level, course stream, and socioeconomic class were important factors to determine the knowledge level, with higher education, science group and high socioeconomic status related to high knowledge level. Knowledge was also significantly associated with religion and sexual activity. Christians, being more sexually active were also more knowledgeable about STIs than their other counterparts. Same is the case with sexually active students who had higher knowledge as compared to students who claimed to be sexually non active. However, both of these groups, in spite of having comparatively better knowledge of STIs are involved in sexual activity. It suggests that the higher knowledge level alone cannot always ensure responsible behavior, as indicated previously. This type of finding has been confirmed by Shapiro, *et al*., 1999, indicating that greater knowledge may be associated with high risk behavior [23].

### Study limitations

There are certain limitations that must be considered in interpreting the results of this study. We did not have a method to verify the claims of students. Also possibility of recall bias or incorrect statement on certain factors such as, age at first intercourse, number of sexual partners, exact age and so forth cannot be ignored. School students from only form 4, form 5 and form 6 have been targeted which may not show the complete picture of school students' awareness and attitude. Further research is required that should also target the students from form 1, form 2 and form 3. Use of self answered questionnaires itself has some limitations such as the difficulties in validating answers, mainly since anonymity could not be fully guaranteed. The findings of this study about awareness and knowledge cannot be generalized to the whole population of students because of small sample size as compared to the total number of students in Pulau Pinang. However, this study provides relevant information for a better understanding of knowledge and sexual behavior among students having different demographic characteristics.

## Conclusions

Malaysia, while developing industrially, also is going through perceptible changes socially and culturally. These changes seem to exert a considerable effect on the already complex nature of the country's young adult population. Knowledge about sexual health and STIs is insufficient among young people of Pulau Pinang who participated in this study although considerable percentage of them is involved in risky sexual behavior which may adversely affect the prevalence of STIs and AIDS in the population of Pulau Pinang. Likewise, problem exists in converting knowledge and positive attitudes into responsible behavior. Little is known on the current status of sexuality education in the schools. Further research on the views of school teachers and students' perception on sexuality education is required in order to implement it effectively so as to fill the knowledge gap.

## Recommendations

Following recommendations are made to improve the awareness of school students:

1. The link between STIs and HIV/AIDS should be reinforced. Emphasizing that STIs increase the likelihood of HIV transmission may increase people's concern about STIs and lead to less risky behavior. Media should also highlight non-HIV STIs.

2. STIs and HIV/AIDS prevention education programmes, seminars and public talks should be conducted regularly. When planning these education programmes, in addition to scientific data on sexual behavior, the socio-cultural and multiracial structure of society and moral norms should also be considered. Efforts should be focused on those segments of population shown to have lower knowledge levels. Educational activities should target parents and teachers as well, which would enable them to play beneficial role in the sexual and reproductive health of young generation.

3. Further detailed research is required to assess the sexual behavior and attitudes of school students; it should also include students belonging to form 1 and form 2 and should be conducted at national level.

## Competing interests

The authors declare that they have no competing interests.

## Authors' contributions

MA conducted the field work and drafted the manuscript and SASS conceived and supervised the project. KA and TMK helped in statistical analysis and manuscript review. All authors read and approved the final manuscript.

## Pre-publication history

The pre-publication history for this paper can be accessed here:

http://www.biomedcentral.com/1471-2458/10/47/prepub

## Supplementary Material

Additional file 1 **Response of students on questions related to knowledge and source of information**. Table showing the details of students' responses on knowledge questions and source of information. Click here for file

Additional file 2 **Important socio-demographic factors for determining sexual behavior**. Table showing the details of socio-demographic factors for determining sexual behavior. Click here for file
