# Correction: Awareness of school students on sexually transmitted infections (STIs) and their sexual behaviour: a cross-sectional study conducted in Pulau Pinang, Malaysia

**DOI:** 10.1186/1471-2458-10-571

**Published:** 2010-09-23

**Authors:** Mudassir Anwar, Syed Azhar S Sulaiman, Keivan Ahmadi, Tahir M Khan

**Affiliations:** 1 School of Pharmacy and Health Sciences, International Medical University, Kuala Lumpur, Malaysia; 2 School of Pharmaceutical Sciences, Universiti Sains Malaysia, Pulau Pinang, Malaysia; 3 School of Pharmacy, Island College of Technology, Sungai Rusa, 11000 Balik Pulau, Pulau Pinang, Malaysia

## Correction

After publication of this work [1], we became aware of a mistake in providing the name of the committee which granted the ethical approval. The approval was obtained from Clinical Research Centre (CRC), Ministry of Health, Malaysia and not from the Social and Behavioural Research Ethics Committee, University Sains Malaysia as mentioned in the original article [1]. Also under the result section it is mistakenly written that "interestingly students who claimed to be sexually active were less knowledgeable (mean score = 11.49 ± 8.710) than those who never had sexual intercourse (mean score = 12.90 ± 8.479)". The correct finding that matches with Table two is "interestingly students who claimed to be sexually active were more knowledgeable (mean score = 12.90 ± 8.479) than those who never had sexual intercourse (mean score = 11.49 ± 8.710)''.

We regret any inconvenience that this inaccurate information might have caused.

## Pre-publication history

The pre-publication history for this paper can be accessed here:

http://www.biomedcentral.com/1471-2458/10/571/prepub
