# Supplementary material for: Awareness of school students on sexually transmitted infections (STIs) and their sexual behavior: a cross-sectional study conducted in Pulau Pinang, Malaysia
Source: BMC Public Health. 2010 Jan 30;10:47. doi: 10.1186/1471-2458-10-47 (PMC2824738; doi:10.1186/1471-2458-10-47)
Supplement: Additional file 1 — Response of students on questions related to knowledge and source of information. Table showing the details of students' responses on knowledge questions and source of information. [file 1471-2458-10-47-S1.DOC]

**Additional File 1:** Response of students on questions related to knowledge and source of information

| N=1139 | **n** | **%** |
| --- | --- | --- |
| **What STIs have you heard of?**  AIDS  Syphilis  Leukemia  Gonorrhoea  Herpes  Rheumatoid arthritis  Chlamydia  Trichomoniasis  Don’t know  **What are some of the symptoms of STIs?**  Itching on genital area  Sores on sexual organs  Painful urination  Discharge from genital area  Feeling of weakness  Genital warts  Body sores  Abdominal pain  Don’t know  **Which of the following do you think are the measures to avoid getting STIs?**  Avoid sexual contact with prostitutes  Use condom during sex  Avoid sexual intercourse  Limit number of sexual partners  Avoid homosexual contact  Sterilize syringes/needles  Non penetrative sex  Take antibiotics prior to sexual intercourse  Don’t know  **Among the following who are at risk of getting STIs?**  Those who have multiple sex partners  Those who receive contaminated blood  Sex workers  Homosexuals, bisexuals  Those who have sexual relations with prostitutes  Drug addicts  Nobody, equal possibilities  Don’t know | 980  381  300  237  87  54  33  13  103  429  417  341  310  249  178  99  512  380  777  612  399  364  335  147  103  62  122  726  682  612  408  354  344  66  132 | 86.0  33.5  26.3  20.8  7.6  4.7  2.9  1.1  9.0  37.7  36.6  29.9  27.2  21.9  15.6  8.7  4.5  33.4  68.2  53.7  35.0  32.0  29.4  12.9  9.0  5.4  10.7  63.7  59.9  53.7  35.8  31.1  30.2  5.8  11.6 |

Table 2 …….continued

|  | **n** | **%** |
| --- | --- | --- |
| **Which of the following do you think are the complications of STIs?**  Increased risk of HIV/AIDS  Increased risk of other infectious conditions  Fertility problems  Some kind of cancer  Don’t know  **STIs can be transmitted by which of the following acts?**  Vaginal sex  Anal intercourse  Oral sex  Kissing  Hugging  Don’t know  **Which of the following organisms do you think can cause STIs?**  Virus  Bacteria  Fungi  Don’t know  **Which of the following sources do you think are effective in providing information on STIs?**  News papers/Magazines  TV shows/Movies  Friends  Internet  Seminars  Family/Parents  Text books  Teachers  No information | 787  377  339  213  208  781  522  357  95  14  241  568  405  118  333  792  585  484  391  218  192  165  10  48 | 69.1  33.1  29.8  18.7  18.3  68.6  45.8  31.3  8.3  1.2  21.2  49.9  35.6  10.4  29.2  69.5  51.4  42.5  34.3  19.1  16.9  14.5  0.9  4.2 |

*Note: Multiple responses were obtained so the collective percentage was not 100%*
