# Supplementary material for: Awareness of school students on sexually transmitted infections (STIs) and their sexual behavior: a cross-sectional study conducted in Pulau Pinang, Malaysia
Source: BMC Public Health. 2010 Jan 30;10:47. doi: 10.1186/1471-2458-10-47 (PMC2824738; doi:10.1186/1471-2458-10-47)
Supplement: Additional file 2 — Important socio-demographic factors for determining sexual behavior. Table showing the details of socio-demographic factors for determining sexual behavior. [file 1471-2458-10-47-S2.DOC]

| N=1139 | **Sexual intercourse**  **No answer Yes No p-value** | **Number of partners**^‡^  **1 2 3 and above p-value** |
| --- | --- | --- |
| **Total**  **Gender**  Male  Female  **Ethnicity**  Malay  Chinese  Indian  Others^†^  **Education level**  Form 4  Form 5  Form 6 | **12 (8) 104 (12.6) 1023 (84.6)**  1 (4.7) 83 (17.5) 390 (77.8)  11 (1.7) 21(3.2) 633 (95.1) 0.003*  9 (1.6) 65 (11.3) 502 (86.8)  3 17 (0.9) 328 (87.1) 0.001*  - 21 (16.1) 190 (83.9)  - 3 (75)^†^ 1 (25)^†^  2 (0.6) 41 (12.1) 296 (87.3)  4 (8.5) 40 (9.6) 373 (81.9) 0.030**  6 (1.6) 23 (6.0) 354 (92.4) | **42 (40.3) 22 (21.5) 40 (38.2)**  31 (37.9) 14 (16.5) 38 (45.6)  10 (46.3) 7 (34.2) 4 (19.5) 0.010*  38 (57.9) 12 (18.0) 15 (24.1)  7 (43.3) 4 (26.7) 5 (30.0) 0.985**  12 (57.4) 4 (16.7) 5 (25.9)  1 (33.3)^†^ - 2 (66.7)^†^  16 (38.2) 11 (27.3) 14 (34.5)  20 (49.1) 4 (11.3) 16 (39.6) 0.709**  10 (41.7) 7 (30.6) 6 (27.7) |

**Additional File 2: Important socio-demographic factors for determining sexual behavior**

Note: Responses are mentioned as **n (%)**

*Significant (p < 0.05) Chi-square test

**Non-significant (p > 0.05) Chi-square test

^†^ Not included in Chi-square test because of small sample size

^‡^ Responses only from those who claimed to be sexually active
